# The Role of Salivary miR-134-3p and miR-15b-5p as Potential Non-invasive Predictors for Not Developing Acute Mountain Sickness

**DOI:** 10.3389/fphys.2019.00898

**Published:** 2019-07-16

**Authors:** He Huang, Huaping Dong, Jianyang Zhang, Xianfeng Ke, Peng Li, Erlong Zhang, Gang Xu, Bingda Sun, Yuqi Gao

**Affiliations:** ^1^Institute of Medicine and Hygienic Equipment for High Altitude Region, College of High Altitude Military Medicine, Army Medical University (Third Military Medical University), Chongqing, China; ^2^Key Laboratory of Extreme Environmental Medicine, Ministry of Education of China, Chongqing, China; ^3^Key Laboratory of High Altitude Medicine, PLA, Chongqing, China; ^4^Department of High Altitude Operational Medicine, College of High Altitude Military Medicine, Army Medical University (Third Military Medical University), Chongqing, China

**Keywords:** hypoxia, high altitude, acute mountain sickness, saliva, microRNA, cerebral tissue oxygenation indices, predictor

## Abstract

**Background:**

Acute mountain sickness (AMS) is a crucial public health problem for high altitude travelers. Discriminating individuals who are not developing (AMS resistance, AMS−) from developing AMS (AMS susceptibility, AMS+) at baseline would be vital for disease prevention. Salivary microRNAs (miRNAs) have emerged as promising non-invasive biomarkers for various diseases. Thus, the aim of our study was to identify the potential roles of salivary miRNAs in identifying AMS− individuals pre-exposed to high altitude. Moreover, as hypoxia is the triggering factor for AMS, present study also explored the association between cerebral tissue oxygenation indices (TOI) and AMS development after exposed to high altitude, which was the complementary aim.

**Methods:**

In this study, 124 healthy men were recruited, and were exposed at simulated high altitude of 4,500 m. Salivary miR-134-3p and miR-15b-5p were measured at baseline (200 m). AMS was diagnosed based on Lake Louise Scoring System at 4,500 m. The measurements of physiological parameters were recorded at both the altitudes.

**Results:**

Salivary miR-134-3p and miR-15b-5p were significantly up-regulated in AMS− individuals as compared to the AMS+ (*p* < 0.05). In addition, the combination of these miRNAs generated a high power for discriminating the AMS− from AMS+ at baseline (AUC: 0.811, 95% CI: 0.731−0.876, *p* < 0.001). Moreover, the value of cerebral TOIs at 4,500 m were significantly higher in AMS− individuals, compared to AMS+ (*p* < 0.01).

**Conclusion:**

Our study reveals for the first time that salivary miR-134-3p and miR-15b-5p can be used as non-invasive biomarkers for predicting AMS− individuals pre-exposed to high altitude.

## Introduction

Acute mountain sickness (AMS) is a prevalent disease among travelers exposed to high altitudes of >2,500 m and presents as a combination of several symptoms, such as headache, dizziness, gastrointestinal symptoms, and fatigue (Roach et al., 2018). The incidence of this disease varies from 16 to 100%, and depends on several factors, such as the speed of ascent, altitude, and individual predisposition (Gaillard et al., 2004; MacInnis et al., 2013; McDevitt et al., 2014; Waeber et al., 2015; Roach et al., 2018). The severe forms of AMS can lead to high altitude cerebral edema in the travelers, having life-threatening consequences (Bartsch and Swenson, 2013; Liu et al., 2017a; Meier et al., 2017). Indeed, AMS has become a crucial public health problem owing to a significant rise in the number of travelers ascending per year (MacInnis et al., 2013; Gonggalanzi et al., 2016, 2017). As such, discriminating individuals who are not developing (AMS resistance, AMS−) from developing AMS (AMS susceptibility, AMS+) pre-exposed to high altitudes would be vital for disease prevention. Presently, the knowledge on AMS resistance and susceptibility is limited to some physiological parameters and gene polymorphisms (Zhou et al., 2004; Koehle et al., 2010; Cochand et al., 2011; Karinen et al., 2012; Kovtun and Voevoda, 2013; Luo et al., 2014; MacInnis and Koehle, 2016; Bailey and Ogoh, 2017; Sutherland et al., 2017; Yasukochi et al., 2018). However, due to the low sensitivity and specificity, their clinical applications are limited (Ding et al., 2011; Song et al., 2013). Thus, there is an unmet need to find a convenient and efficient biomarker for identifying AMS− individuals at baseline.

MicroRNA (miRNA) are 21∼23-nucleotide long, single-stranded, non-coding RNA, which are an important class of gene-modulators for various physiological and disease processes, such as cell cycle, growth, development, differentiation, apoptosis, and inflammatory response (Zhou et al., 2016; Wang et al., 2017). In the recent years, miRNA has been found to be stably expressed in saliva, thus being proven as a convenient and non-invasive biomarker for cancer, Sjögren’s syndrome, concussion symptoms, and aging (Weber et al., 2010; Xie et al., 2013, 2015; Machida et al., 2015; Alhasan et al., 2016; Greither et al., 2017; Johnson et al., 2018). Importantly, our recent study on plasma miRNA array has demonstrated that 16 miRNAs were up-regulated and 15 were down-regulated in AMS− individuals at baseline (Liu et al., 2017b). Specifically, our pilot study involved the evaluation of the salivary expression levels of the five topmost up-regulated miRNAs, which led to the identification of miR-134-3p and miR-15b-5p as abundantly expressed in both whole saliva and its supernatant.

Based on these findings, we hypothesized that salivary miR-134-3p and miR-15b-5p may aid in discriminating between AMS− and AMS+. Therefore, the aims of the present study were to examine whether salivary miR-134-3p and miR-15b-5p could be identified as non-invasive biomarkers for predicting AMS− individuals at baseline, and to evaluate their discriminatory powers. Moreover, as hypoxia is the triggering factor for AMS, the present study also explored the association between cerebral tissue oxygenation indices (TOI) and AMS development after exposed to high altitude.

## Materials and Methods

### Participants

Participants were recruited according to the inclusion and exclusion criteria. The inclusion criteria involved healthy individuals, without primary residence at an elevation of ≥1,000 m. Exclusion criteria were listed as follows: individuals with history of travel to an elevation of >2,500 m in the last 2 years, cardio-cerebrovascular diseases, respiratory diseases, kidney diseases, liver diseases, and neuropsychological diseases. In total, 124 healthy Chinese men aged 20–23 years were recruited in the present study.

This study protocol was approved by the Third Military Medical University Ethics Committee, China, meeting with the requirements of the Declaration of Helsinki, and all individuals signed informed consent forms before entry.

### Study Procedures

**FIGURE 1:**
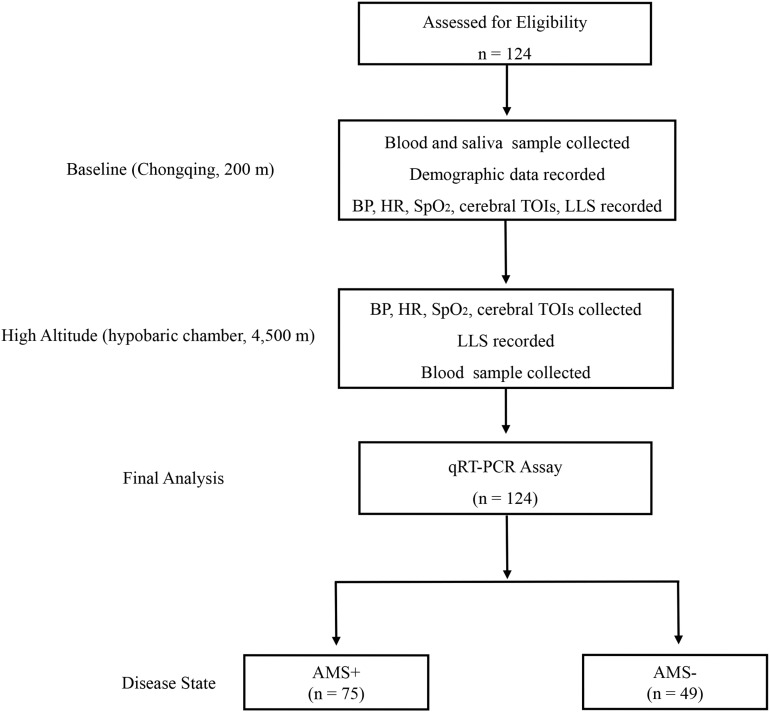
Trial flow diagram. AMS+, participant with acute mountain sickness (AMS); AMS–, participant without AMS; qRT-PCR, quantitative reverse-transcription polymerase chain reaction; LLS, Lake Louise Score; BP, blood pressure; HR, heart rate; SpO_2_, pulse oxygen saturation; TOI, tissue oxygenation indices.

Following the methods of previous studies (Burtscher et al., 2014; Broessner et al., 2016), all participants were exposed at the simulated high altitude of 4,500 m [hypobaric chamber (Feng Lei, Guizhou, China), temperature: 23–25°C, humidity: 23–27%] for 12 h [the time of ascending from baseline (200 m) to 4,500 m is 40 min]. At baseline (8:00 a.m.), blood sample, saliva sample, demographic data, and physiological parameters were collected from the participants. After a 12 h-exposure at 4,500 m (8:40 p.m.), the participants were subjected to diagnosis of AMS, measurement of physiological parameters, and collection of blood sample (Figure 1). During the investigation, participants were provided with the same diet (no coffee, tea, or alcohol drinks), and required to avoid strenuous physical activity. Security assurances, accompanying physicians, immediate evacuation, and medical treatment were available.

### Clinical Data Collection and AMS Diagnosis

At baseline, a self-reported questionnaire was used to record the demographic data (i.e., age, body mass index [BMI], smoking, and drinking history). After the 12 h-exposure at 4,500 m, our accompanying physicians evaluated the AMS of individuals. It was assessed based on Lake Louise Scoring System, which comprises a questionnaire and a scorecard that determine severity (Roach et al., 2018). As per the diagnostic criteria, the score was calculated as a combination of headache, dizziness, fatigue, and gastrointestinal symptoms (Roach et al., 2018). Finally, the participants with headache and a score ≥3 points, were diagnosed with AMS (AMS+). Participants who had no headache or a score <3 were classified as AMS−, i.e., without AMS.

### Physiological Measurements

The basic physiological parameters, including heart rate (HR), pulse oxygen saturation (SpO_2_), diastolic blood pressure (DBP), and systolic blood pressure (SBP), were measured by our accompanying doctors with a sphygmomanometer (HEM-6200, OMRON, China) and a pulse oximeter (NONIN-9550, Nonin Onyx, United States). Cerebral TOI for the left and right brain were measured using continuous wave near-infrared spectroscopy (NIRO-200NX, Hamamatsu Photonics, Japan). In detail, two sensors were attached to each participant, one on the left and the other on the right forehead (approximately 2.5 cm above the eyebrows) and held gently with a double-sided tape. The sampling frequency and time duration were 50 Hz and 5 min, respectively. TOI was computed using a spatially resolved spectroscopy algorithm and defined as oxygenated hemoglobin as a percentage of total hemoglobin, which represents the mixed oxygenation level of the cerebral compartments. Finally, the mean value of TOI within 5 min was used in the present study. The participants rested for 30 min before the evaluation. In total, our tests have been conducted at baseline (8:00 a.m.) and at 4,500 m (8:40 p.m.), separately. Moreover, all the instruments were validated by a medical device engineer.

### Samples Collection and White Blood Cell Count Measurements

At baseline, up to 5 mL of saliva sample was obtained in a 50-mL centrifuge tube from each individual. In order to stimulate glandular salivary flow, the cotton swab with 2% citric acid solution was provided, which is used to touch the bilateral posterior lateral surfaces of the tongue (5 s every 30 s) (Xie et al., 2013). Then, a total of 2 mL of saliva was removed from the tube as whole saliva sample. The remaining 3 mL of saliva sample was centrifuged at a speed of 3,000 × *g* for 15 min under 4°C, to spin down the exfoliated cells. After that, the supernatant was further centrifuged (12,000 × *g*, 10 min, 4°C) to completely remove the cellular components. Finally, the samples (whole saliva and supernatant saliva) were aliquoted into RNase/DNase free Eppendorf tubes and stored at −80°C until assay. Based on the previous methods of Xie et al. (2013, 2015), using citric acid in cotton swab can stimulate glandular salivary flow, but does not alter the results. At baseline and after the exposure at 4,500 m, venous blood samples were collected from the participants by qualified nurses using EDTA-coated tubes and standard procedures. The blood samples were stored at 4°C until further testing. Then, the white blood cell count (WBC) was analyzed using the AU-2700 analyzers (Olympus, Tokyo, Japan) and commercial reagents.

### RNA Extraction and qRT-PCR Assay

Before RNA was isolated from the whole saliva or supernatant saliva samples, 3.5 μl of the working solution of synthetic *Caenorhabditis elegans* miRNA, cel-miR-39 (Qiagen, Valencia, CA, United States), was added as a control. Then, similar to a previous study (Gao et al., 2014), miRNeasy extraction kit (Qiagen, Valencia, CA, United States) was used to isolate the total RNA from samples based on the instruction of the manufacturer. For qRT-PCR assay, the Bulge-Loop^*TM*^ miRNA qRT-PCR Starter Kit (including primers) (Ribobio, Guangzhou, China) was used for reverse transcription and iQ^*TM*^5 Real-Time PCR Detection System (Bio-Rid, United States) was used for performing quantitative real-time PCR. Data was analyzed according to the 2^–Δ*CT*^ method.

### MIRNA Target Computational Analysis

In order to explore the biological functions of miR-134-3p and miR-15b-5p, we predicted the target genes of these miRNAs by employing microT-CDS v5.0, which is the new version of the microserver and has been significantly enhanced with an improved target prediction algorithm (Vlachos et al., 2012; Paraskevopoulou et al., 2013; Alhasan et al., 2016). Then, these target genes were enriched into gene ontology (GO) biological processes using the software DIANA-miRPath v3.0 (Vlachos et al., 2015).

### Statistical Analysis

Shapiro–Wilk’s test was used for calculating the normality of all data. Then, the normally distributed data was expressed as mean ± standard deviation, while the non-normally distribution was expressed as median (interquartile range). For the normally distributed data, the independent *t*-test was used to compare the differences whereas for the non-normally distributed data, the Mann–Whitney *U* test was employed. Spearman’s correlation and Pearson’s correlation were carried out for analyzing relationships between the AMS severity and variables. Univariate logistic regression was used to identify the protectors for AMS. Then, a multivariant logistic regression with enter method was used to confirm independent protectors. Receiver operating characteristic (ROC) curve was applied for each miRNA and the combination. Areas under the ROC curve (AUC) and 95% confidence interval (CI) were calculated to evaluate the power of miRNAs for distinguishing AMS− from AMS+ groups. Statistical analyses were performed with IBM SPSS Statistics 19 (SPSS, Chicago, IL, United States), and MedCalc Statistical Software version 15.4 (MedCalc Software bvba, Ostend, Belgium). *p* < 0.05 was considered statistically significant.

## Results

### Demographic Data and Clinical Characteristics of Participants

In the present study, the incidence of AMS is 60.5% (75 out of 124). There was no significant difference between AMS+ and AMS− groups in age (21.44 ± 0.66 vs. 21.55 ± 0.78, *p* = 0.664), BMI (22.43 ± 2.72 vs. 22.27 ± 1.86, *p* = 0.500), smoking (66.7 vs. 65.3%, *p =* 0.888), and drinking rate (76.0 vs. 77.5%, *p* = 0.843). Moreover, in comparison with the AMS− group, the AMS+ group had higher LLS (5.57 ± 2.08 vs. 1.69 ± 0.98, *p* < 0.001, Table 1). For all baseline parameters, there was no significant difference between the two groups. Regarding the physiological parameters at 4,500 m, SpO_2_ and the cerebral TOIs, were significantly higher in AMS− group, as compared to the AMS+ group (all *p* < 0.05, Table 2).

**TABLE 1:** Characteristics of participants.

	**AMS+ (75)**	**AMS− (49)**	***p* value**
**Demographic data**			
Age (year)			
Mean	21.44 ± 0.66	21.55 ± 0.78	0.664
Range	20–23	20–23	
BMI (kg/m^2^)	22.43 ± 2.72	22.27 ± 1.86	0.500
Smoker (yes)	50 (66.7%)	32 (65.3%)	0.888
Drinker (yes)	57 (76.0%)	38 (77.5%)	0.843
**AMS severity**			
LLS	5.57 ± 2.08	1.69 ± 0.98	<0.001^∗∗∗^

*AMS+, participant with acute mountain sickness (AMS); AMS−, participant without AMS; BMI, body mass index; LLS, Lake Louise Score. ^∗∗∗^Means LLS are significantly different between AMS+ and AMS− groups at 4,500 m with *p* value < 0.001. Data was expressed as means ± standard deviations.*

**TABLE 2:** Difference of physiological parameters between AMS+ and AMS− groups.

	**AMS+**		**AMS−**	
	**Baseline**	**4,500 m**	**Baseline**	**4,500 m**
SBP (mmHg)	118.67 ± 9.50	115.01 ± 13.78	118.32 ± 10.15	116.73 ± 10.90
DBP (mmHg)	70.78 ± 7.45	68.17 ± 11.05	70.79 ± 7.46	72.00 ± 8.15
HR (beat/min)	66.98 ± 9.50	89.03 ± 11.49	66.14 ± 8.59	86.67 ± 12.98
SpO_2_ (%)	98.04 ± 1.13	80.05 ± 6.66	98.32 ± 0.90	82.71 ± 5.69^*^
Left brain TOI (%)	72.35 ± 4.36	60.90 ± 5.13	71.47 ± 4.58	63.43 ± 4.89^∗∗^
Right brain TOI (%)	72.21 ± 4.98	60.39 ± 4.50	70.67 ± 5.27	62.02 ± 4.06^∗∗^
Average brain TOI (%)	72.00 ± 5.50	60.65 ± 4.52	71.07 ± 4.04	62.72 ± 3.84^∗∗^

AMS+, participant with acute mountain sickness (AMS); AMS−, participant without AMS; BP, blood pressure; SBP, systolic BP; DBP, diastolic BP; HR, heart rate; SpO_2_, pulse oxygen saturation; TOI, tissue oxygenation indices. ^*^Means parameters are significantly different between AMS+ and AMS− groups at 4,500 m with *p* value < 0.05; ^∗∗^Means parameters are significantly different between AMS+ and AMS− groups at 4,500 m with *p* value < 0.01.

### Differences in Salivary miR-134-3p and miR-15b-5p Between AMS+ and AMS− Groups

**FIGURE 2:**
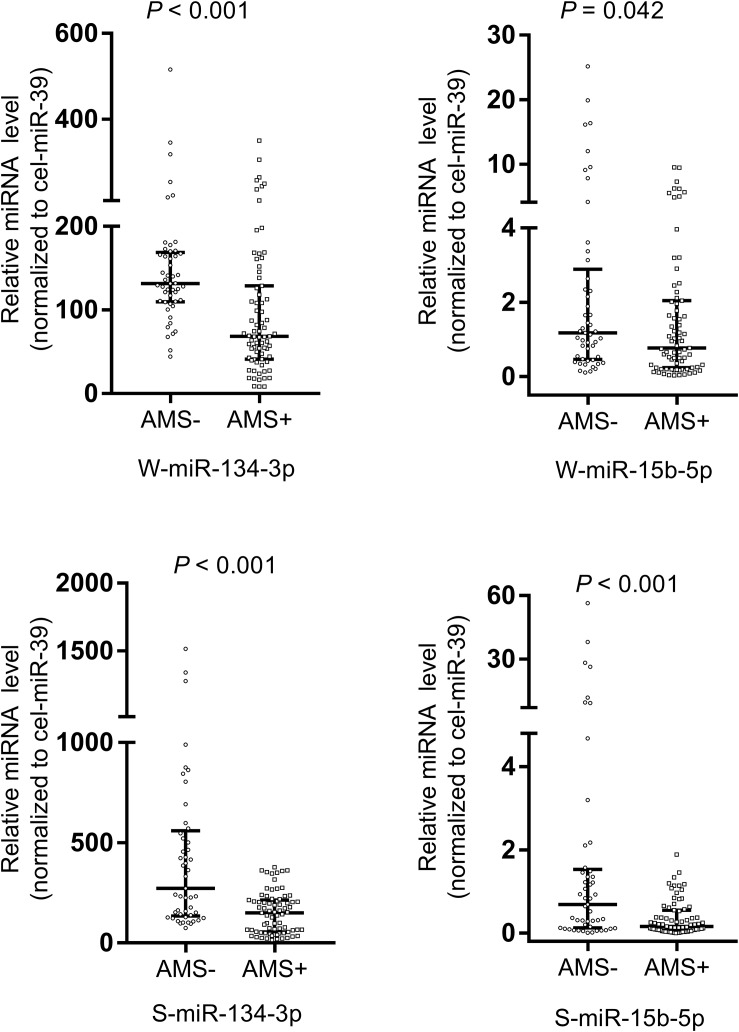
Salivary miR-134-3p and miR-15b-5p were different between acute mountain sickness (AMS+) and non-acute mountain sickness (AMS–) groups. These miRNAs were significantly down-regulated in AMS+ group (*n* = 75) compared with AMS– group (*n* = 49). A non-parametric test (Mann–Whitney *U* test) was employed to compare miRNAs in these two independent groups. Data was expressed as median (interquartile range). The expression level of W-miR-134-3p, S-miR-134-3p and S-miR-15b-5p were significantly different between AMS+ and AMS– groups at baseline with *p* value < 0.01. The expression level of W-miR-15b-5p were significantly different between AMS+ and AMS– groups at baseline with *p* value < 0.05. S-miR-134-3p: miR-134-3p of supernatant saliva; S-miR-15b-5p: miR-15b-5p of supernatant saliva; W-miR-134-3p: miR-134-3p of whole saliva; W-miR-15b-5p: miR-15b-5p of whole saliva.

Upon employing cel-miR-39 as the normalization control, the results of qRT-PCR assay revealed that miR-134-3p (*p* < 0.001) and miR-15b-5p (*p* < 0.05) of whole saliva (W-miR-134-3p and W-miR-15b-5p) were significantly up-regulated in the AMS− group as compared to the AMS+ group. Similarly, miR-134-3p and miR-15b-5p of supernatant saliva (S-miR-134-3p and S-miR-15b-5p) were also significantly up-regulated (all *p* < 0.001, Figure 2).

### Salivary MiRNA Signature for Discriminating AMS− From AMS+ Individuals

**FIGURE 3:**
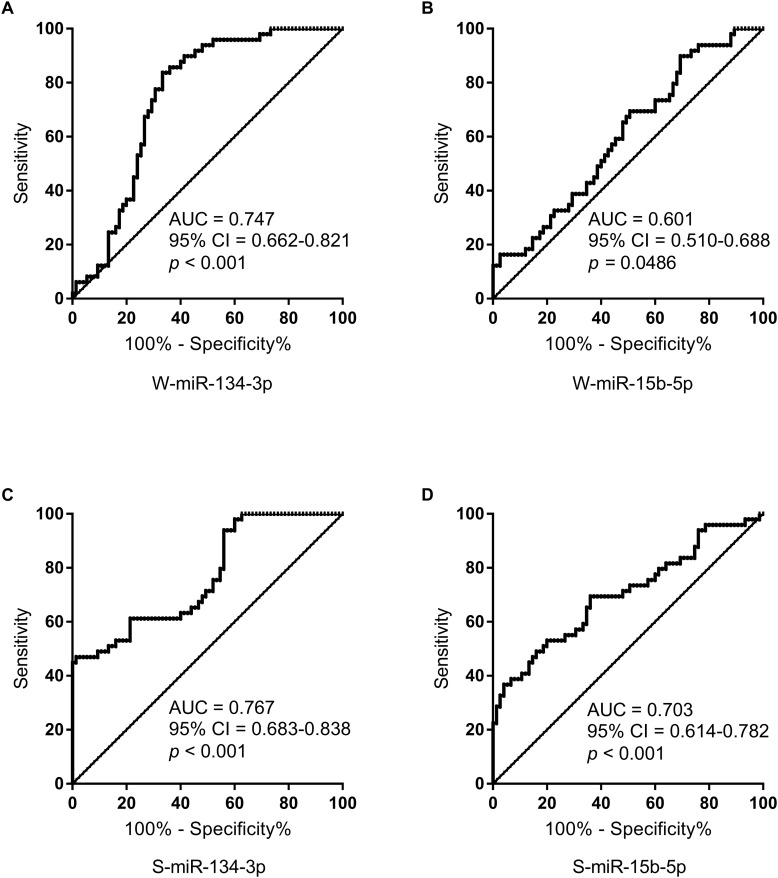
ROC curve analysis for salivary miRNAs to discriminate AMS– from AMS+ groups. **(A)** ROC curve analysis for W-miR-134-3p. **(B)** ROC curve analysis for W-miR-15b-5p. **(C)** ROC curve analysis for S-miR-134-3p. **(D)** ROC curve analysis for S-miR-15b-5p. AUC, area under curve; CI, confidence interval.

Receiver operating characteristic curves were computed to evaluate the power of miRNAs for discriminating AMS− from AMS+ individuals. The AUC of W-miR-134-3p, W-miR-15b-5p, S-miR-134-3p, and S-miR-15b-5p was 0.747, 0.601, 0.767, and 0.703, respectively (Figures 3A–D).

**FIGURE 4:**
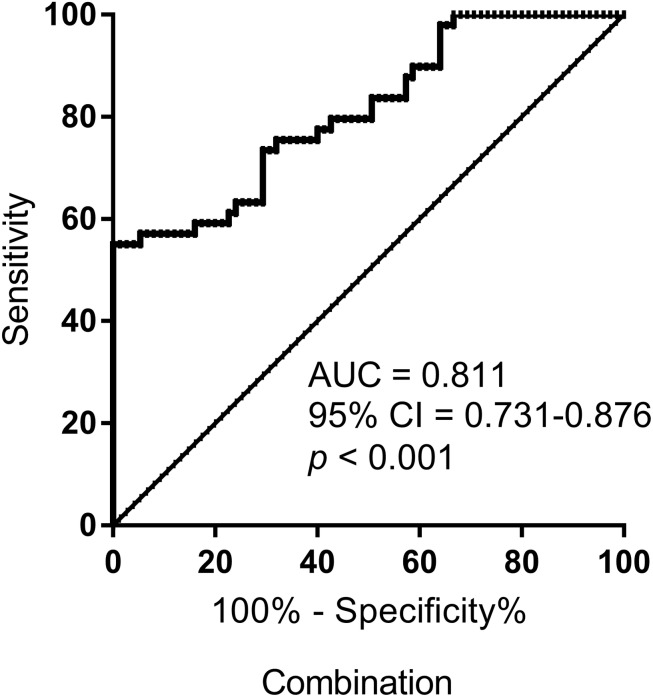
ROC curve analysis for the combination of S-miR-134-3p and S-miR-15b-5p to discriminate AMS– from AMS+ groups. AUC, area under curve; CI, confidence interval.

To improve the accuracy of identification, we performed ROC curves for the combination of S-miR-134-3p and S-miR-15b-5p, using logistic regression analysis. Notably, the combination resulted in a robustly increased AUC (0.811), leading to a unique signature for identifying AMS− individuals (Figure 4).

### Salivary miR-134-3p and miR-15b-5p as Protectors for AMS Development

The univariate logistic regression analyses revealed that higher expression levels of W-miR-15b-5p, S-miR-15b-5p, and the combination (S-miR-134-3p + S-miR-15b-5p) were protectors for AMS development (all *p* < 0.05). Furthermore, multivariate logistic regression analyses showed that higher expression levels of S-miR-15b-5p at baseline were independent protectors for AMS (all *p* < 0.05, Table 3). Moreover, higher expression levels of W-miR-134-3p, S-miR-134-3p might be possible protectors for AMS, which need to be studied in the future.

**TABLE 3:** Univariate and multivariate logistic regression for salivarymiRNAs at baseline (*n* = 124).

			**95% CI**		
**Risk factors**	**β-coefficient**	**Odds ratio**	**Lower**	**Upper**	***p* value**
**Univariate logistic regression**					
W-miR-134-3p	−0.009	0.991	0.985	0.996	0.001^∗∗^
W-miR-15b-5p	−0.123	0.884	0.790	0.989	0.032^*^
S-miR-134-3p	−0.007	0.993	0.990	0.996	<0.001^∗∗∗^
S-miR-15b-3p	−1.090	0.336	0.162	0.699	0.004^∗∗^
Combination	−5.447	0.004	0.001	0.032	<0.001^∗∗∗^
**Multivariate logistic regression**					
W-miR-134-3p	−0.006	0.994	0.988	0.999	0.044^*^
S-miR-134-3p	−0.006	0.994	0.991	0.998	0.001^∗∗^
S-miR-15b-3p	−1.029	0.357	0.149	0.856	0.021^*^

CI, confidence interval; S-miR-134-3p, miR-134-3p of supernatant saliva; S-miR-15b-5p, miR-15b-5p of supernatant saliva; W-miR-134-3p, miR-134-3p of whole saliva; W-miR-15b-5p, miR-15b-5p of whole saliva. Combination: S-miR-134-3p+ S-miR-15b-5p. For univariate and multivariate logistic regression, the dependent variable is AMS development (1 = AMS+, 0 = AMS−). ^*^p value < 0.05; ^∗∗^*p* value < 0.01; ^∗∗∗^*p* value < 0.001.

### Biological Process Regulated by miR-134-3p and miR-15b-5p

The GO enrichment analysis exhibited that miR-134-3p and miR-15b-5p could regulate biological processes associated with the inflammatory response, such as the innate immune response (GO:0045087), toll-like receptor 10 signaling pathway (GO:0034166), toll-like receptor TLR1:TLR2 signaling pathway (GO:0038123), toll-like receptor TLR6:TLR2 signaling pathway (GO:0038124), toll-like receptor 5 signaling pathway (GO:0034146), toll-like receptor 9 signaling pathway (GO:0034162), toll-like receptor 2 signaling pathway (GO:0034134), toll-like receptor 4 signaling pathway (GO:0034142), TRIF-dependent toll-like receptor signaling pathway (GO:0035666), MyD88-independent toll-like receptor signaling pathway (GO:0002756), toll-like receptor 3 signaling pathway (GO:0034138), and stress-activated MAPK cascade (GO:0051403) (Supplementary Table 1).

## Discussion

This is the first study, so far, to report that (i) salivary miR-134-3p and miR-15b-5p were significantly up-regulated in AMS− individuals at baseline; (ii) both salivary miR-134-3p and miR-15b-5p served as non-invasive biomarkers for discriminating AMS− from AMS+ individuals pre-exposed to high altitudes; and (iii) AMS− individuals were featured with higher value of cerebral TOIs.

Nowadays, the biological relevance of miRNAs in body fluid circulation is regarded as a global, hormone-like functional molecule, which might regulate gene expression across tissues (Turchinovich et al., 2013; Yamakuchi and Hashiguchi, 2018). Here, we found that the expression values of salivary miR-134-3p and miR-15b-5p in the AMS− individuals, were significantly higher in the AMS− than the AMS+ individuals. Their target genes were enriched in toll-like receptor signaling pathway and stress-activated MAPK pathway, which are major signaling pathways for modulating inflammation (Mohan and Gupta, 2018; Koga et al., 2019).

Previous studies affirm that increased vascular permeability due to hypoxia-induced inflammation is involved in the pathophysiology of AMS development (Julian et al., 2011; Boos et al., 2016; Luks et al., 2017). Recently, our study found that the concentration of inflammatory cytokines, including IL-1β, IL-6, and TNF-α, are positively correlated to AMS severity (Song et al., 2016). Moreover, another important point to note from our recent study regarding transcriptome analysis is that immune and inflammatory responses are overrepresented in AMS+ individuals, but not in AMS− (Liu et al., 2017a). Intriguingly, dexamethasone, the first line treatment for AMS, has proven to be quite effective in producing an anti-inflammatory response via inhibiting toll-like receptor signaling pathway and stress-activated MAPK pathway (Chuang et al., 2017; Speer et al., 2018). In this regard, the higher expression value of miR-134-3p and miR-15b-5p in AMS− individuals suppresses the expression of genes involved in toll-like receptor signaling pathway and stress-activated MAPK pathway post-transcriptionally, and in turn repress inflammatory response. Thus, the dampened inflammatory response via miRNAs may be a biological factor of AMS− individuals who reveal a higher expression level of miR-134-3p and miR-15b-5p.

The immune system is a highly regulated system to several extrinsic factors including environmental stress (Muhie et al., 2013). The concept that hypoxia can induce inflammation has also gained credence in some recent studies (Eltzschig and Carmeliet, 2011). Our group has also revealed recently that hypoxia could exacerbate the inflammatory response via stimulating toll-like receptor four signaling pathway in rats (Wu et al., 2018). Consistently, several studies in mice have also proposed that hypoxia-induced inflammatory response could result in an enhanced vascular permeability, which is associated with the pathophysiology of AMS development (Singh et al., 2016; Zhou et al., 2017; Gong et al., 2018). Interestingly, the present study revealed that the AMS− individuals presented with less severe hypoxemia (higher SpO_2_ and cerebral TOIs) than the AMS+ individuals at high altitude, which was consisted with previous studies (Basnyat, 2014; Guo et al., 2014; Mandolesi et al., 2014; Harrison et al., 2016; Leichtfried et al., 2016). Taken together, hypoxia could be a driver of the inflammatory response, the less severe hypoxemia and the higher expression level of miR-134-3p and miR-15b-5p could alleviate inflammatory response in synergy, thus contributing to AMS prevention.

### Limitations

We demonstrated for the first time, two novel non-invasive salivary miRNAs for identifying AMS− individuals. However, only the young Chinese men were included in this study because they are a part of the population that most frequently travels to high altitudes. Moreover, AMS was diagnosed in the hypobaric chamber, and the rate of ascent was faster than that for a regular travel to high-altitude regions, which may lead to a higher disease incidence. In addition, our participants were all slim, which perhaps could have skewed the results. Therefore, further investigations in high altitude regions, larger sample sizes, different genders, age, race, and BMI, should be studied to affirm the results obtained.

## Conclusion

In this study, we report for the first time, two non-invasive biomarkers, salivary miR-134-3p and miR-15b-5p, for identifying AMS− individuals pre-exposed to high altitudes. Furthermore, the combination of miR-134-3p and miR-15b-5p may hold a great promise in becoming an important non-invasive tool for AMS prevention in the future.

## Data Availability

All datasets generated for this study are included in the manuscript and/or the Supplementary Files.

## Ethics Statement

This study was carried out in accordance with the recommendations of the Third Military Medical University Ethics Committee, with written informed consent from all subjects. This study protocol was approved by the Third Military Medical University Ethics Committee, China, meeting with the requirements of the Declaration of Helsinki, and all individuals signed informed consent forms before entry.

## Author Contributions

YG conceived and designed the study. HD and XK oversaw the laboratory analyses. HH provided the overall supervision of the study and drafted the manuscript. GX, PL, and BS did the statistical analysis and contributed the laboratory experiments. JZ, EZ, HD, and XK contributed to sample and data collections. All authors contributed to the interpretation of results, critical revision of the manuscript, and approved the final manuscript. YG is the guarantor.

## Conflict of Interest Statement

The authors declare that the research was conducted in the absence of any commercial or financial relationships that could be construed as a potential conflict of interest.

## References

[B1] Alhasan A. H. Scott A. W. Wu J. J. Feng G. Meeks J. J. Thaxton C. S. (2016). Circulating microRNA signature for the diagnosis of very high-risk prostate cancer. *Proc. Natl. Acad. Sci. U.S.A.* 113 10655–10660. 10.1073/pnas.1611596113 27601638 PMC5035901

[B2] Bailey D. M. Ogoh S. (2017). Heterogeneous regulation of cerebral blood flow in hypoxia; implications for dynamic cerebral autoregulation and susceptibility to acute mountain sickness. *Exp. Physiol.* 102:383. 10.1113/EP086144 28247477

[B3] Bartsch P. Swenson E. R. (2013). Clinical practice: acute high-altitude illnesses. *N. Engl. J. Med.* 368 2294–2302. 10.1056/NEJMcp1214870 23758234

[B4] Basnyat B. (2014). Pro: pulse oximetry is useful in predicting acute mountain sickness. *High Alt. Med. Biol.* 15 440–441. 10.1089/ham.2014.1045 25531458 PMC4273179

[B5] Boos C. J. Woods D. R. Varias A. Biscocho S. Heseltine P. Mellor A. J. (2016). High altitude and acute mountain sickness and changes in circulating endothelin-1, interleukin-6, and interleukin-17a. *High Alt. Med. Biol.* 17 25–31. 10.1089/ham.2015.0098 26680502

[B6] Broessner G. Rohregger J. Wille M. Lackner P. Ndayisaba J. P. Burtscher M. (2016). Hypoxia triggers high-altitude headache with migraine features: a prospective trial. *Cephalalgia* 36 765–771. 10.1177/0333102415610876 26487467

[B7] Burtscher M. Wille M. Menz V. Faulhaber M. Gatterer H. (2014). Symptom progression in acute mountain sickness during a 12-hour exposure to normobaric hypoxia equivalent to 4500 m. *High Alt. Med. Biol.* 15 446–451. 10.1089/ham.2014.1039 25341048

[B8] Chuang T. Y. Cheng A. J. Chen I. T. Lan T. Y. Huang I. H. Shiau C. W. (2017). Suppression of LPS-induced inflammatory responses by the hydroxyl groups of dexamethasone. *Oncotarget* 8 49735–49748. 10.18632/oncotarget.17683 28537905 PMC5564803

[B9] Cochand N. J. Wild M. Brugniaux J. V. Davies P. J. Evans K. A. Wise R. G. (2011). Sea-level assessment of dynamic cerebral autoregulation predicts susceptibility to acute mountain sickness at high altitude. *Stroke* 42 3628–3630. 10.1161/STROKEAHA.111.621714 21960569

[B10] Ding H. Liu Q. Hua M. Ding M. Du H. Zhang W. (2011). Polymorphisms of hypoxia-related genes in subjects susceptible to acute mountain sickness. *Respiration* 81 236–241. 10.1159/000322850 21242666

[B11] Eltzschig H. K. Carmeliet P. (2011). Hypoxia and inflammation. *N. Engl. J. Med.* 364 656–665. 10.1056/NEJMra0910283 21323543 PMC3930928

[B12] Gaillard S. Dellasanta P. Loutan L. Kayser B. (2004). Awareness, prevalence, medication use, and risk factors of acute mountain sickness in tourists trekking around the Annapurnas in Nepal: a 12-year follow-up. *High Alt. Med. Biol.* 5 410–419. 10.1089/ham.2004.5.410 15671630

[B13] Gao S. Chen L. Y. Wang P. Liu L. M. Chen Z. (2014). MicroRNA expression in salivary supernatant of patients with pancreatic cancer and its relationship with ZHENG. *Biomed. Res. Int.* 2014:756347. 10.1155/2014/756347 25126577 PMC4122139

[B14] Gong G. Yin L. Yuan L. Sui D. Sun Y. Fu H. (2018). Ganglioside GM1 protects against high altitude cerebral edema in rats by suppressing the oxidative stress and inflammatory response via the PI3K/AKT-Nrf2 pathway. *Mol. Immunol.* 95 91–98. 10.1016/j.molimm.2018.02.001 29428576

[B15] Gonggalanzi Labasangzhu Bjertness E. Wu T. Stigum H. Nafstad P. (2017). Acute mountain sickness, arterial oxygen saturation and heart rate among Tibetan students who reascend to Lhasa after 7 years at low altitude: a prospective cohort study. *BMJ Open* 7:e016460. 10.1136/bmjopen-2017-016460 28698346 PMC5726117

[B16] Gonggalanzi Labasangzhu Nafstad P. Stigum H. Wu T. Haldorsen O. D. (2016). Acute mountain sickness among tourists visiting the high-altitude city of Lhasa at 3658 m above sea level: a cross-sectional study. *Arch. Public Health* 74:23. 10.1186/s13690-016-0134-z 27252854 PMC4888367

[B17] Greither T. Vorwerk F. Kappler M. Bache M. Taubert H. Kuhnt T. (2017). Salivary miR-93 and miR-200a as post-radiotherapy biomarkers in head and neck squamous cell carcinoma. *Oncol. Rep.* 38 1268–1275. 10.3892/or.2017.5764 28677748

[B18] Guo G. Zhu G. Sun W. Yin C. Ren X. Wang T. (2014). Association of arterial oxygen saturation and acute mountain sickness susceptibility: a meta-analysis. *Cell Biochem. Biophys.* 70 1427–1432. 10.1007/s12013-014-0076-4 24965166

[B19] Harrison M. F. Anderson P. J. Johnson J. B. Richert M. Miller A. D. Johnson B. D. (2016). Acute mountain sickness symptom severity at the south pole: the influence of self-selected prophylaxis with acetazolamide. *PLoS One* 11:e0148206. 10.1371/journal.pone.0148206 26848757 PMC4744068

[B20] Johnson J. J. Loeffert A. C. Stokes J. Olympia R. P. Bramley H. Hicks S. D. (2018). Association of salivary microRNA changes with prolonged concussion symptoms. *JAMA Pediatr.* 172 65–73. 10.1001/jamapediatrics.2017.3884 29159407 PMC5833519

[B21] Julian C. G. Subudhi A. W. Wilson M. J. Dimmen A. C. Pecha T. Roach R. C. (2011). Acute mountain sickness, inflammation, and permeability: new insights from a blood biomarker study. *J. Appl. Physiol.* 111 392–399. 10.1152/japplphysiol.00391.2011 21636566 PMC3154693

[B22] Karinen H. M. Uusitalo A. Vaha-Ypya H. Kahonen M. Peltonen J. E. Stein P. K. (2012). Heart rate variability changes at 2400 m altitude predicts acute mountain sickness on further ascent at 3000-4300 m altitudes. *Front. Physiol.* 3:336. 10.3389/fphys.2012.00336 22969727 PMC3431006

[B23] Koehle M. S. Guenette J. A. Warburton D. E. (2010). Oximetry, heart rate variability, and the diagnosis of mild-to-moderate acute mountain sickness. *Eur. J. Emerg. Med.* 17 119–122. 10.1097/MEJ.0b013e32832fa099 19641462

[B24] Koga Y. Tsurumaki H. Aoki-Saito H. Sato M. Yatomi M. Takehara K. (2019). Roles of cyclic AMP response element binding activation in the ERK1/2 and p38 MAPK signalling pathway in central nervous system, cardiovascular system, osteoclast differentiation and mucin and cytokine production. *Int. J. Mol. Sci.* 20:E1346. 10.3390/ijms20061346 30884895 PMC6470985

[B25] Kovtun L. T. Voevoda M. I. (2013). Susceptibility to hypoxia and breathing control changes after short-term cold exposures. *Int. J. Circumpolar Health* 72:21574. 10.3402/ijch.v72i0.21574 23967415 PMC3748441

[B26] Leichtfried V. Basic D. Burtscher M. Gothe R. M. Siebert U. Schobersberger W. (2016). Diagnosis and prediction of the occurrence of acute mountain sickness measuring oxygen saturation–independent of absolute altitude? *Sleep Breath.* 20 435–442. 10.1007/s11325-015-1195-x 26032284

[B27] Liu B. Chen J. Zhang L. Gao Y. Cui J. Zhang E. (2017a). IL-10 dysregulation in acute mountain sickness revealed by transcriptome analysis. *Front. Immunol.* 8:628. 10.3389/fimmu.2017.00628 28611780 PMC5447681

[B28] Liu B. Huang H. Wu G. Xu G. Sun B. D. Zhang E. L. (2017b). A Signature of circulating microRNAs predicts the susceptibility of acute mountain sickness. *Front. Physiol.* 8:55. 10.3389/fphys.2017.00055 28228730 PMC5296306

[B29] Luks A. M. Swenson E. R. Bartsch P. (2017). Acute high-altitude sickness. *Eur. Respir. Rev.* 26:160096. 10.1183/16000617.0096-2016 28143879 PMC9488514

[B30] Luo Y. Wang Y. Lu H. Gao Y. (2014). ‘Ome’ on the range: update on high-altitude acclimatization/adaptation and disease. *Mol. Biosyst.* 10 2748–2755. 10.1039/c4mb00119b 25099339

[B31] Machida T. Tomofuji T. Ekuni D. Maruyama T. Yoneda T. Kawabata Y. (2015). MicroRNAs in salivary exosome as potential biomarkers of aging. *Int. J. Mol. Sci.* 16 21294–21309. 10.3390/ijms160921294 26370963 PMC4613253

[B32] MacInnis M. J. Carter E. A. Freeman M. G. Pandit B. P. Siwakoti A. Subedi A. (2013). A prospective epidemiological study of acute mountain sickness in nepalese pilgrims ascending to high altitude (4380 m). *PLoS One* 8:e75644. 10.1371/journal.pone.0075644 24130729 PMC3794000

[B33] MacInnis M. J. Koehle M. S. (2016). Evidence for and against genetic predispositions to acute and chronic altitude illnesses. *High Alt. Med. Biol.* 17 281–293. 10.1089/ham.2016.0024 27500591

[B34] Mandolesi G. Avancini G. Bartesaghi M. Bernardi E. Pomidori L. Cogo A. (2014). Long-term monitoring of oxygen saturation at altitude can be useful in predicting the subsequent development of moderate-to-severe acute mountain sickness. *Wilderness Environ. Med.* 25 384–391. 10.1016/j.wem.2014.04.015 25027753

[B35] McDevitt M. McIntosh S. E. Rodway G. Peelay J. Adams D. L. Kayser B. (2014). Risk determinants of acute mountain sickness in trekkers in the Nepali Himalaya: a 24-year follow-up. *Wilderness Environ. Med.* 25 152–159. 10.1016/j.wem.2013.12.027 24864065

[B36] Meier D. Collet T. H. Locatelli I. Cornuz J. Kayser B. Simel D. L. (2017). Does this patient have acute mountain sickness?: the rational clinical examination systematic review. *JAMA* 318 1810–1819. 10.1001/jama.2017.16192 29136449

[B37] Mohan S. Gupta D. (2018). Crosstalk of toll-like receptors signaling and Nrf2 pathway for regulation of inflammation. *Biomed. Pharmacother.* 108 1866–1878. 10.1016/j.biopha.2018.10.019 30372892

[B38] Muhie S. Hammamieh R. Cummings C. Yang D. Jett M. (2013). Transcriptome characterization of immune suppression from battlefield-like stress. *Genes Immun.* 14 19–34. 10.1038/gene.2012.49 23096155 PMC3564018

[B39] Paraskevopoulou M. D. Georgakilas G. Kostoulas N. Vlachos I. S. Vergoulis T. Reczko M. (2013). DIANA-microT web server v5.0: service integration into miRNA functional analysis workflows. *Nucleic Acids Res.* 41 W169–W173. 10.1093/nar/gkt393 23680784 PMC3692048

[B40] Roach R. C. Hackett P. H. Oelz O. Bartsch P. Luks A. M. MacInnis M. J. (2018). The 2018 lake louise acute mountain sickness score. *High Alt. Med. Biol.* 19 4–6. 10.1089/ham.2017.0164 29583031 PMC6191821

[B41] Singh D. P. Nimker C. Paliwal P. Bansal A. (2016). Ethyl 3,4-dihydroxybenzoate (EDHB): a prolyl hydroxylase inhibitor attenuates acute hypobaric hypoxia mediated vascular leakage in brain. *J. Physiol. Sci.* 66 315–326. 10.1007/s12576-015-0429-9 26649730 PMC10717431

[B42] Song H. Ke T. Luo W. J. Chen J. Y. (2013). Non-high altitude methods for rapid screening of susceptibility to acute mountain sickness. *BMC Public Health* 13:902. 10.1186/1471-2458-13-902 24079477 PMC3852617

[B43] Song T. T. Bi Y. H. Gao Y. Q. Huang R. Hao K. Xu G. (2016). Systemic pro-inflammatory response facilitates the development of cerebral edema during short hypoxia. *J. Neuroinflammation* 13:63. 10.1186/s12974-016-0528-4 26968975 PMC4788817

[B44] Speer E. M. Dowling D. J. Xu J. Ozog L. S. Mathew J. A. Chander A. (2018). Pentoxifylline, dexamethasone and azithromycin demonstrate distinct age-dependent and synergistic inhibition of TLR- and inflammasome-mediated cytokine production in human newborn and adult blood in vitro. *PLoS One* 13:e0196352. 10.1371/journal.pone.0196352 29715306 PMC5929513

[B45] Sutherland A. Freer J. Evans L. Dolci A. Crotti M. Macdonald J. H. (2017). MEDEX 2015: heart rate variability predicts development of acute mountain sickness. *High Alt. Med. Biol.* 18 199–208. 10.1089/ham.2016.0145 28418725

[B46] Turchinovich A. Samatov T. R. Tonevitsky A. G. Burwinkel B. (2013). Circulating miRNAs: cell-cell communication function? *Front. Genet.* 4:119. 10.3389/fgene.2013.00119 23825476 PMC3695387

[B47] Vlachos I. S. Kostoulas N. Vergoulis T. Georgakilas G. Reczko M. Maragkakis M. (2012). DIANA miRPath v.2.0: investigating the combinatorial effect of microRNAs in pathways. *Nucleic Acids Res.* 40 W498–W504. 10.1093/nar/gks494 22649059 PMC3394305

[B48] Vlachos I. S. Zagganas K. Paraskevopoulou M. D. Georgakilas G. Karagkouni D. Vergoulis T. (2015). DIANA-miRPath v3.0: deciphering microRNA function with experimental support. *Nucleic Acids Res.* 43 W460–W466. 10.1093/nar/gkv403 25977294 PMC4489228

[B49] Waeber B. Kayser B. Dumont L. Lysakowski C. Tramer M. R. Elia N. (2015). Impact of study design on reported incidences of acute mountain sickness: a systematic review. *High Alt. Med. Biol.* 16 204–215. 10.1089/ham.2015.0022 26230550

[B50] Wang Y. Yang Z. Le W. (2017). Tiny but mighty: promising roles of microRNAs in the diagnosis and treatment of parkinson’s disease. *Neurosci. Bull.* 33 543–551. 10.1007/s12264-017-0160-z 28762215 PMC5636733

[B51] Weber J. A. Baxter D. H. Zhang S. Huang D. Y. Huang K. H. Lee M. J. (2010). The microRNA spectrum in 12 body fluids. *Clin. Chem.* 56 1733–1741. 10.1373/clinchem.2010.147405 20847327 PMC4846276

[B52] Wu G. Xu G. Chen D. W. Gao W. X. Xiong J. Q. Shen H. Y. (2018). Hypoxia exacerbates inflammatory acute lung injury via the toll-like receptor 4 signaling pathway. *Front. Immunol.* 9:1667. 10.3389/fimmu.2018.01667 30083155 PMC6064949

[B53] Xie Z. Chen G. Zhang X. Li D. Huang J. Yang C. (2013). Salivary microRNAs as promising biomarkers for detection of esophageal cancer. *PLoS One* 8:e57502. 10.1371/journal.pone.0057502 23560033 PMC3613402

[B54] Xie Z. Yin X. Gong B. Nie W. Wu B. Zhang X. (2015). Salivary microRNAs show potential as a noninvasive biomarker for detecting resectable pancreatic cancer. *Cancer Prev. Res.* 8 165–173. 10.1158/1940-6207.CAPR-14-0192 25538087

[B55] Yamakuchi M. Hashiguchi T. (2018). Endothelial cell aging: how miRNAs contribute? *J. Clin. Med.* 7:170. 10.3390/jcm7070170 29996516 PMC6068727

[B56] Yasukochi Y. Nishimura T. Motoi M. Watanuki S. (2018). Association of EGLN1 genetic polymorphisms with SpO2 responses to acute hypobaric hypoxia in a Japanese cohort. *J. Physiol. Anthropol.* 37:9. 10.1186/s40101-018-0169-7 29625625 PMC5889538

[B57] Zhou Q. Gao Y. Huang Q. (2004). Predictive effect of lung functional determination of the population susceptible to acute mountain sickness. *Med. J. Natl. Defending Forces in Northwest China* 25 13–15.

[B58] Zhou S. Ding F. Gu X. (2016). Non-coding RNAs as emerging regulators of neural injury responses and regeneration. *Neurosci. Bull.* 32 253–264. 10.1007/s12264-016-0028-7 27037691 PMC5563772

[B59] Zhou Y. Huang X. Zhao T. Qiao M. Zhao X. Zhao M. (2017). Hypoxia augments LPS-induced inflammation and triggers high altitude cerebral edema in mice. *Brain Behav. Immun.* 64 266–275. 10.1016/j.bbi.2017.04.013 28433745

