# Supplementary material for: The Role of Salivary miR-134-3p and miR-15b-5p as Potential Non-invasive Predictors for Not Developing Acute Mountain Sickness
Source: Front Physiol. 2019 Jul 16;10:898. doi: 10.3389/fphys.2019.00898 (PMC6646415; doi:10.3389/fphys.2019.00898)
Supplement: Supplementary file 1 [file Table_1.DOCX]

**Table 1.** GO enrichment for the target genes of miR-134-3p and miR-15b-5p.

| **GO ID** | **GO Category** | ***p* value** | **NO. of genes** | **NO. of miRNAs** |
| --- | --- | --- | --- | --- |
| GO:0045087 | innate immune response | 2.90E-02 | 46 | 2 |
| GO:0034166 | toll-like receptor 10 signaling pathway | 6.77E-04 | 9 | 2 |
| GO:0038123 | toll-like receptor TLR1:TLR2 signaling pathway | 1.04E-03 | 9 | 2 |
| GO:0038124 | toll-like receptor TLR6:TLR2 signaling pathway | 1.04E-03 | 9 | 2 |
| GO:0034146 | toll-like receptor 5 signaling pathway | 1.60E-03 | 9 | 2 |
| GO:0034162 | toll-like receptor 9 signaling pathway | 3.21E-03 | 9 | 2 |
| GO:0034134 | toll-like receptor 2 signaling pathway | 8.45E-03 | 9 | 2 |
| GO:0034142 | toll-like receptor 4 signaling pathway | 4.68E-02 | 9 | 2 |
| GO:0035666 | TRIF-dependent toll-like receptor signaling pathway | 9.43E-03 | 8 | 2 |
| GO:0002756 | MyD88-independent toll-like receptor signaling pathway | 1.92E-02 | 8 | 2 |
| GO:0034138 | toll-like receptor 3 signaling pathway | 4.10E-02 | 8 | 2 |
| GO:0051403 | stress-activated MAPK cascade | 2.20E-02 | 7 | 2 |

GO: gene ontology; NO.: number.

The GO biological processes related to inflammatory response are listed in this table.
